# Neoadjuvant Chemotherapy With Selpercatinib for Locally Advanced *RET* Fusion-Positive Papillary Thyroid Carcinoma: A Case Report

**DOI:** 10.1155/crie/6676471

**Published:** 2025-06-03

**Authors:** Mei Kadoya, Katsuhiko Masudo, Hiroyuki Ito, Yoichiro Okubo, Yohei Miyagi, Hiroyuki Hayashi, Hiroyuki Iwasaki

**Affiliations:** ^1^Department of Endocrine Surgery, Kanagawa Cancer Center, 2-3-2 Nakao, Asahi-ku, Yokohama 241-8515, Kanagawa, Japan; ^2^Department of Respiratory Surgery, Kanagawa Cancer Center, 2-3-2 Nakao, Asahi-ku, Yokohama 241-8515, Kanagawa, Japan; ^3^Department of Pathology, Kanagawa Cancer Center, 2-3-2 Nakao, Asahi-ku, Yokohama 241-8515, Kanagawa, Japan; ^4^Department of Pathology, Yokohama Municipal Citizen's Hospital, 1–1 Mitsuzawanishimachi, Kanagawa-Ku, Yokohama 221-0855, Kanagawa, Japan

## Abstract

A 65-year-old male presented to our hospital with a complaint of a left cervical mass. The left supraclavicular lymph node was enlarged, measuring 77 mm, and biopsy results confirmed metastasis of papillary thyroid carcinoma (PTC). The left supraclavicular lymph node extended to the upper mediastinum and invaded the internal jugular and subclavian veins, with suspicion of common carotid and subclavian artery invasion. Surgical resection was deemed infeasible. The Oncomine Dx Target Test system, a gene panel test using a next-generation sequencer, of the metastatic lymph node was positive for *RET* fusion (*CCDC6-RET*), and selpercatinib treatment was initiated. After 4 months, the tumor reduced in size, and surgery was performed. The postoperative course was uneventful, with ongoing follow-up. This case is a successful case of neoadjuvant chemotherapy for *RET* fusion-positive PTC with local regional progression.

## 1. Introduction

Selpercatinib, a selective RET inhibitor, has shown high efficacy and safety for *RET* mutation-positive medullary thyroid carcinoma (MTC) and *RET* fusion-positive thyroid carcinoma in the LIBRETTO-001 trial [1]. The efficacy of neoadjuvant chemotherapy for locally advanced thyroid cancer has been recently reported [2, 3]. However, only a few studies on neoadjuvant chemotherapy with selpercatinib have been conducted [4, 5]. We here report a case of initially unresectable papillary thyroid carcinoma (PTC) that became resectable following neoadjuvant chemotherapy with selpercatinib.

## 2. Case Presentation

**Figure 1:**
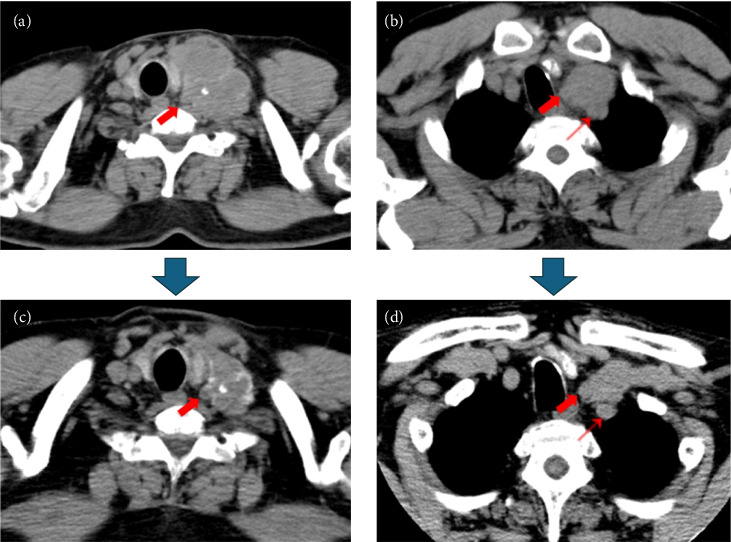
Computed tomography findings before and after neoadjuvant chemotherapy with selpercatinib. The left supraclavicular mass invading the left common carotid artery (➡) and left subclavian artery (→) before the neoadjuvant chemotherapy (a, b). The tumor shows shrinkage upon treatment, and the major axis shows reduced diameter from 77 to 58 mm. Furthermore, the tumor has receded from the left common carotid and left subclavian arteries (c, d).

**Figure 2:**
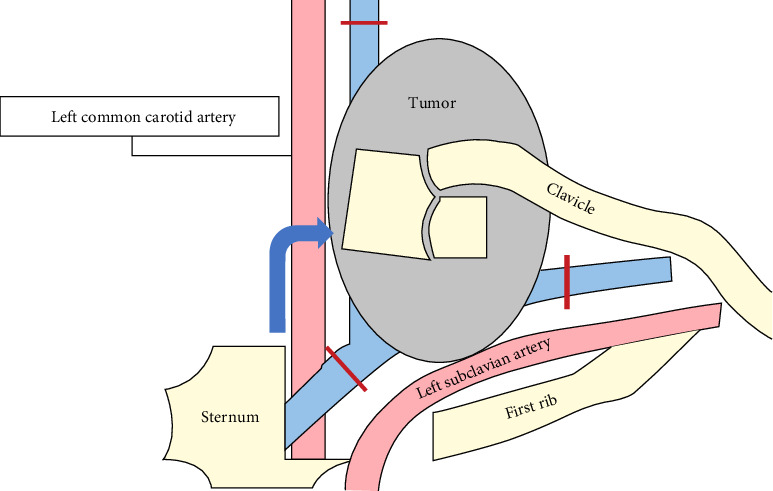
Surgical procedure image. An L-shaped incision of the manubrium and resection of the ventricular part of the first costal cartilage are performed, and the sternoclavicular joint and clavicle are flipped outward.

**Figure 3:**
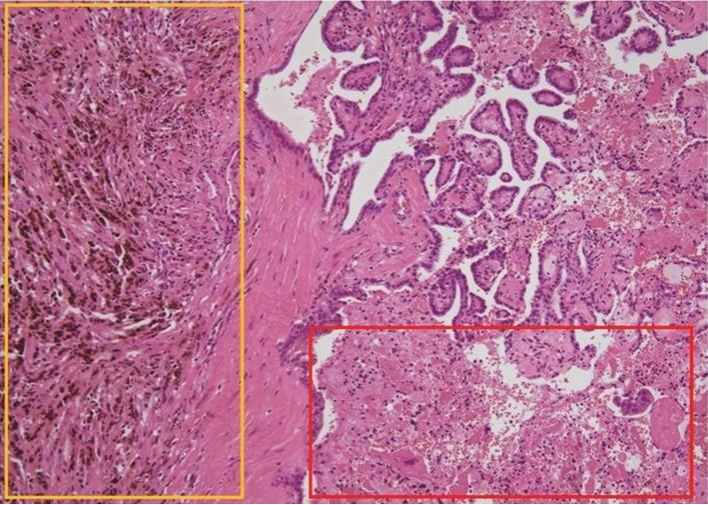
Surgical pathology following neoadjuvant chemotherapy with selpercatinib. In the left supraclavicular lymph node, papillary thyroid carcinoma cells forming papillary structures are still present, along with areas of fibrosis, hemosiderin-laden macrophages (orange box), coagulative necrosis, and inflammatory cells (red box), confirming the therapeutic effect of selpercatinib.

**Figure 4:**
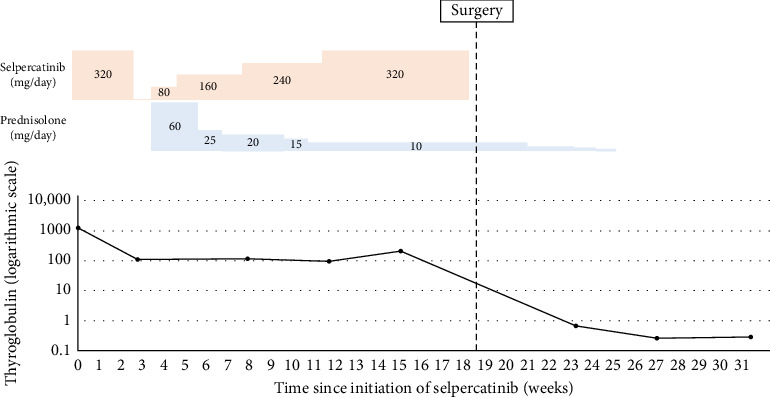
Treatment and clinical course following selpercatinib initiation.

A 65-year-old male patient presented to our hospital with a complaint of a left cervical mass. He had no family history of thyroid cancer or radiation exposure. His anamnesis revealed bronchial asthma and eczema. A hard mass on the left supraclavicular area was revealed on physical examination. Computed tomography (CT) revealed a left thyroid lobe nodule with calcification and a 77 mm-sized lymph node metastasis extending from the left supraclavicular area to the upper mediastinum (Figure 1). No evidence of distant metastasis was observed. Thyroid-stimulating hormone and thyroglobulin levels in the blood were 2.039 (normal range: 0.61–4.23) µIU/mL and 1240 (normal range: 0–35.1) ng/mL, respectively, whereas antithyroglobulin antibody levels were below the detection threshold. Left supraclavicular lymph node biopsy results confirmed the diagnosis of PTC. Left supraclavicular lymph node biopsy revealed PTC, leading to a diagnosis of clinical T2N1bM0 Stage II PTC according to the Union for International Cancer Control (UICC) 8th edition. The left supraclavicular lymph node invaded the internal jugular and subclavian veins, with strong suspicion of common carotid and subclavian artery invasion. Multidisciplinary evaluation determined incomplete surgical resectability. The Oncomine Dx Target Test system, a gene panel test using a next-generation sequencer, of the metastatic lymph node was positive for *RET* fusion (*CCDC6-RET*), and selpercatinib treatment was initiated. However, after 17 days, the patient developed fever, arthralgia, and thrombocytopenia and was diagnosed with hypersensitivity (Grade 2, Common Terminology Criteria for Adverse Events version 5.0). Therefore, selpercatinib was discontinued for 1 week. The left neck mass significantly increased after 1-week drug withdrawal. Treatment was reinitiated with a dose reduction to 80 mg/day, along with concurrent prednisolone administration. In addition, hyperuricemia (Grade 1), hypercalcemia (Grade 1), and diarrhea (Grade 2) developed as adverse events of selpercatinib. At 4 months following selpercatinib initiation, the left supraclavicular lymph node had reduced in size (Figure 1). CT revealed a slight separation between the tumor and the left common carotid and left subclavian arteries, based on which it was determined that the arteries could be preserved and complete tumor resection could be achieved. After holding selpercatinib administration for 3 days, surgery was performed, including total thyroidectomy, left neck dissection, and left internal jugular vein, left subclavian vein, left vagus nerve, and left phrenic nerve resection. The tumor was easily dissectible from the left subclavian artery, but it adhered to the left common carotid artery, so adventitial excision of the left common carotid artery was performed. The left common carotid and left subclavian arteries were preserved, and R1 resection was achieved, indicating gross tumor removal. As shown in the figures, an L-shaped incision of the manubrium and resection of the first costal cartilage are performed, and the sternoclavicular joint and clavicle are flipped outward (Figure 2). Postoperatively, although the patient developed left upper limb edema for 1 week, hoarseness of voice, left phrenic nerve paralysis, and left Horner's syndrome, no laryngeal edema or dysphagia was observed. Wound healing progressed uneventfully; therefore, the patient was discharged on postoperative day 7. The pathological examination revealed a highly calcified mass measuring 22 mm × 15 mm × 24 mm in the left thyroid lobe on gross inspection. Microscopically, fibrous tissues with calcification and ossification, with focal residual tumor cells exhibiting attenuated nuclear features of PTC (e.g., nuclear grooves and optically clear nuclei), predominantly comprised the tumor lesion. In the left supraclavicular lymph node, PTC cells forming papillary structures were still observed, along with areas of coagulative necrosis, fibrosis, inflammatory cells, hemosiderin-laden macrophages, and foamy cells, confirming the therapeutic effect of selpercatinib (Figure 3). The left supraclavicular lymph node metastasis showed pathological invasion into the internal jugular vein, whereas no evident invasion was noted in the subclavian vein or the adventitia of the common carotid artery. The final pathological diagnosis was pT2N1bM0 Stage II PTC (UICC 8th edition). At 3 months postoperatively, no signs of local recurrence were noted, and selpercatinib readministration was not required. CT revealed no evidence of recurrent tumors or distant metastases. The thyroglobulin level decreased from 214 ng/mL preoperatively to 0.28 ng/mL postoperatively (Figure 4), indicating no evidence of disease. Further, radioiodine ablation was planned.

## 3. Discussion

Selpercatinib, which was approved in Japan in February 2022, is a selective RET inhibitor that has shown high efficacy and safety for *RET* mutation-positive MTC and *RET* fusion-positive thyroid carcinoma. Only a few studies on neoadjuvant approach with selpercatinib have been conducted. In this case, the left supraclavicular lymph node metastasis had invaded the common carotid and subclavian arteries, making complete resection challenging. Although multikinase inhibitors, including lenvatinib, were also considered as treatment options, genetic testing results revealed *RET* fusion positivity; therefore, selpercatinib treatment was initiated owing to the expected high efficacy and safety. In this case, continuing the systemic therapy was made a viable option owing to selpercatinib treatment effectiveness. In the present case, no evidence of distant metastases was observed, and a good prognosis could be expected when thyroid and left cervical lymph node metastases could be resected. Therefore, we opted to switch to surgical treatment. In cases of locally advanced PTC, histopathological examination may reveal the presence of undifferentiated components. In such cases, the addition of postoperative external beam radiotherapy or the resumption of treatment with selpercatinib may be considered. Neoadjuvant chemotherapy followed by surgery is associated with various issues, including delayed wound healing and bleeding. However, selpercatinib has minimal activity against VEGFR2 [6], and its short half-life (31.5 h) renders it appropriate for neoadjuvant therapeutic applications. In surgeries performed during selpercatinib therapy, drug withdrawal pre- and postoperatively is recommended to enable tissue recovery and healing; however, the optimal duration for the drug withdrawal remains unclear. Studies on neoadjuvant chemotherapy with selpercatinib for MTC have reported surgical completion without complications after holding selpercatinib administration for 3 days [4, 5]. In the present case, the left neck mass significantly increased after a 1-week drug withdrawal owing to hypersensitivity; therefore, the preoperative drug withdrawal was limited to 3 days. Complications, including delayed wound healing and bleeding, were not observed.

The treatment outcomes for *RET* fusion-positive PTC with local progression may be improved by this approach of neoadjuvant RET-specific inhibitor followed by surgery.

In conclusion, we reported the case of locally advanced PTC that became resectable after treatment with selpercatinib. Neoadjuvant chemotherapy with selpercatinib for locally advanced *RET* fusion-positive PTC may result in complete tumor resection and enhanced treatment outcomes. To assess the safety and efficacy of this approach, further research is needed.

## Data Availability

All the data generated or analyzed during this study are included in this published article.
